# Loss of His-bundle and Right Ventricular Septal Capture Following Transcatheter Aortic Valve Replacement—A Case Report

**DOI:** 10.19102/icrm.2023.14022

**Published:** 2023-02-15

**Authors:** Abdullah Asreb, Joon Ahn

**Affiliations:** ^1^Internal Medicine Residency, Northeast Georgia Medical Center, Gainesville, GA, USA; ^2^Cardiac Electrophysiology, Northeast Georgia Medical Center, Gainesville, GA, USA

**Keywords:** Complete heart block, His bundle, left bundle branch block, transcatheter aortic valve replacement

## Abstract

Conduction abnormalities following transcatheter aortic valve replacement (TAVR) are common. High-grade atrioventricular block (AVB) and new-onset left bundle branch block remain the most reported. These often require the placement of a permanent pacemaker (PPM). His-bundle (HB) pacing is increasingly being utilized as the preferred mode of ventricular pacing due to its more physiologic ventricular activation. In this case report, we present a case of a patient who developed loss of HB capture and experienced an increase in the local right ventricular (RV) capture threshold after TAVR that led to unrecognized intermittent loss of ventricular capture and symptoms. An 80-year-old man with severe aortic stenosis presented with symptomatic bradycardia due to typical atrial flutter (AFL) with a high-grade AVB and an underlying right bundle branch block. He underwent placement of a dual-chamber PPM (Medtronic, Inc., Minneapolis, MN, USA) with a HB pacing lead. HB mapping demonstrated a normal H–V interval, and the lead was fixated with non-selective HB capture. The R-waves measured 2.8 mV, the pacing impedance was 544 Ω, and the non-selective HB and local RV capture threshold was 0.5 V @ 1 ms. He underwent AFL ablation, and his atrial leads were normal. He subsequently underwent successful TAVR with a 29-mm Sapien 3 valve (Edwards Lifesciences, Irvine, CA, USA). Post-TAVR, PPM interrogation showed a loss of HB capture with a left bundle paced QRS morphology. Following discharge, he presented with stroke-like symptoms and was noted to have intermittent loss of RV capture with complete heart block (CHB) and a slow ventricular escape rhythm. PPM interrogation revealed an elevated pacing threshold, and his RV output was gradually increased to a maximum output of 7.5 V @ 1.5 ms. He also developed a fever and was found to have enterococcal bacteremia. Transesophageal echocardiography demonstrated vegetations on his prosthetic valve and pacemaker lead, without a perivalvular abscess. He underwent explantation of the pacemaker system and insertion of a temporary PPM. After intravenous antibiotic therapy with negative blood cultures, he underwent re-implantation of a new right-sided dual-chamber PPM, and an RV pacing lead was placed into the RV outflow tract. HB pacing is becoming the preferred mode of physiologic ventricular pacing. This case illustrates the potential risks of the TAVR procedure in patients with existing HB pacing leads. We observed a loss of HB capture and the development of CHB due to traumatic injury to the HB distal to the HB pacing lead after TAVR placement together with an increase in the local RV capture threshold. The depth of TAVR placement is an important aspect of the TAVR procedure that determines the risk of developing CHB and may also affect the HB and local RV pacing thresholds post-procedure.

## Introduction

Transcatheter aortic valve replacement (TAVR) has revolutionized the management of severe aortic stenosis (AS) as a safe and feasible alternative for patients with severe symptomatic AS in whom the surgical risk is elevated. Conduction abnormalities may occur following TAVR due to the proximity of the atrioventricular (AV) conduction system to the aortic cusps and left ventricular outflow tract (LVOT). The mechanism is related to a direct mechanical injury or an indirect insult through perivalvular inflammation or ischemia.^1^ The risk of developing significant conduction abnormalities is increased when there are pre-existing conduction defects, such as AV nodal conduction block or right bundle branch block (RBBB). New-onset left bundle branch block (LBBB) and high-grade AV block (AVB) are the most reported complications, with an incidence rate of 4%–60%.^1^ In many cases, these complications often require the placement of a permanent pacemaker (PPM). The incidence of PPM placement following TAVR is estimated to be around 2.3%–36.1% according to a recent meta-analysis.^2^ Risk factors for PPM after TAVR include pre-existing RBBB, depth of the valve implant, short membranous septum,^3^ and over-sizing of the implant.^4^ Physiologic pacing therapies, which include His-bundle (HB) pacing and left bundle branch area pacing (LBBAP), are increasingly becoming the preferred methods of pacing in patients who require a high percentage of right ventricular (RV) pacing. The potential benefits are due to physiologic ventricular activation by pacing directly into the His–Purkinje conduction system, which would avoid dyssynchronous ventricular activation and help prevent heart failure, worsening mitral regurgitation, and pacing-induced cardiomyopathy.^5–7^

Malfunctioning of a HB pacemaker due to the loss of HB capture and changes in local septal RV capture thresholds following TAVR have not been described. In this case report, we present the case of a patient who developed a loss of HB capture and had a significantly increased local RV capture threshold after successful TAVR implantation that led to unrecognized intermittent loss of ventricular capture with an eventual need for lead extraction and re-implantation to an alternative RV location.

## Case presentation

**Figure 1:**
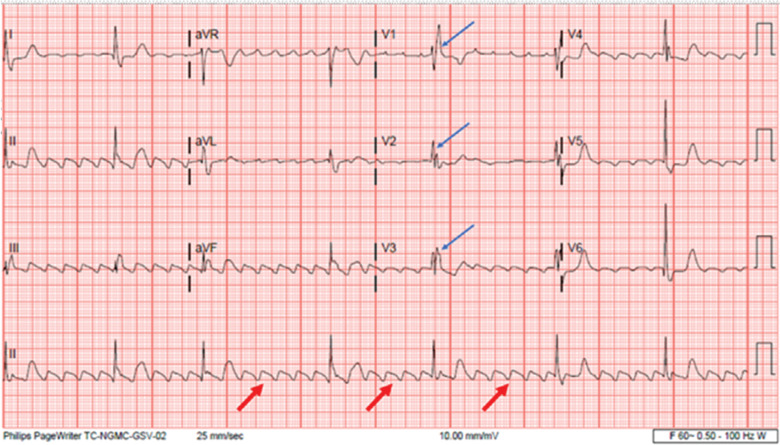
Admission electrocardiogram revealing typical atrial flutter (red arrows) with marked bradycardia (heart rate, 40 bpm) and right bundle branch block (blue arrows).

**Figure 2:**
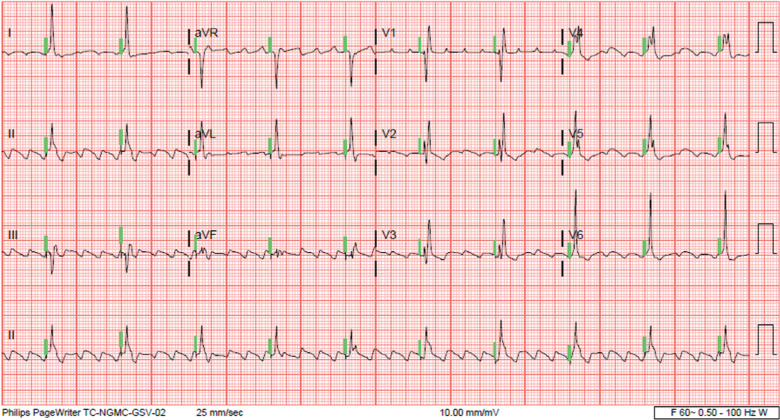
Non-selective His-bundle capture (green lines) and improved heart rates following successful implantation of a Medtronic dual-chamber permanent pacemaker.

An 80-year-old man with a medical history of paroxysmal atrial fibrillation, coronary artery disease, chronic kidney disease, and severe AS presented with dizziness and light-headedness that started a few days prior to his presentation. His laboratory evaluation revealed normal hemoglobin levels, a white blood cell count of 9.8 g/dL, a mildly elevated creatinine level of 1.83 mg/dL, and mild hypokalemia with a K^+^ concentration of 3 mmol/L. His initial electrocardiogram (ECG) showed marked bradycardia due to typical atrial flutter (AFL) with a slow ventricular response as well as baseline RBBB **(Figure 1)**. Given his symptomatic bradycardia, the patient underwent placement of a dual-chamber PPM with an HB pacing lead (3830 SelectSecure; Medtronic, Minneapolis, MN, USA). During implantation, HB mapping demonstrated a normal H–V interval of 54 ms. The lead was fixated at the site with demonstration of non-selective HB capture during pace-mapping. The R-waves measured 2.8 mV, the pacing impedance was 544 Ω, and the non-selective HB capture threshold was 0.5 V @ 1 ms **(Figure 2)**. A follow-up device interrogation 8 days later revealed a non-selective HB capture threshold of 0.75 V @ 1 ms and a selective HB capture threshold of 0.5 V @ 1 ms.

**Figure 3:**
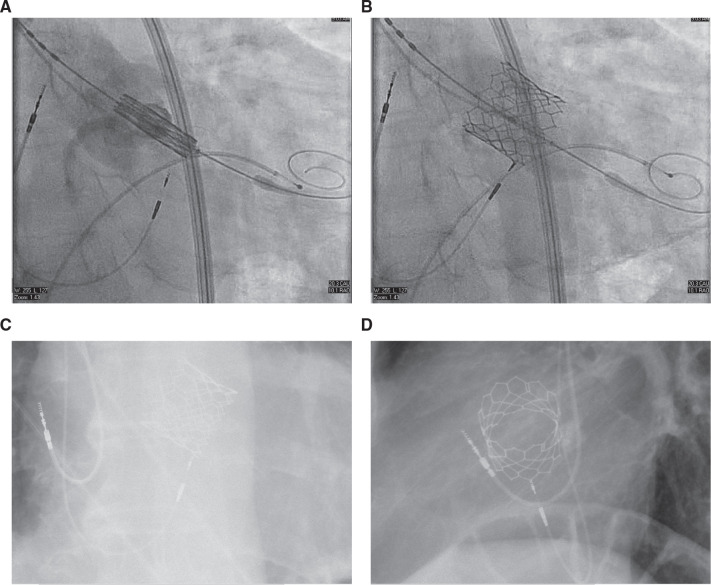
**A–D:** Successful transcatheter aortic valve replacement implantation. Notice the proximity of the prosthetic aortic valve to the pacemaker lead.

The patient’s symptoms improved and he was discharged home. Four months later, he underwent successful cavotricuspid isthmus ablation of isthmus-dependent AFL. Post-ablation, his underlying rhythm was a second-degree AVB in a type I pattern. His atrial lead parameters were found to be normal, and the HB capture thresholds remained stable. He continued to have symptoms attributed to severe AS and subsequently underwent a successful TAVR procedure with a 29-mm Sapien 3 valve (Edwards Lifesciences, Irvine, CA, USA) 8 months after pacemaker implantation **(Figure 3)**.

**Figure 4:**
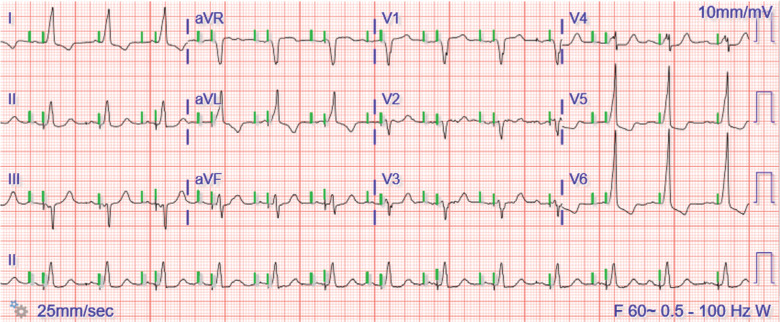
Atrioventricular pacing with loss of His bundle capture.

**Figure 5:**
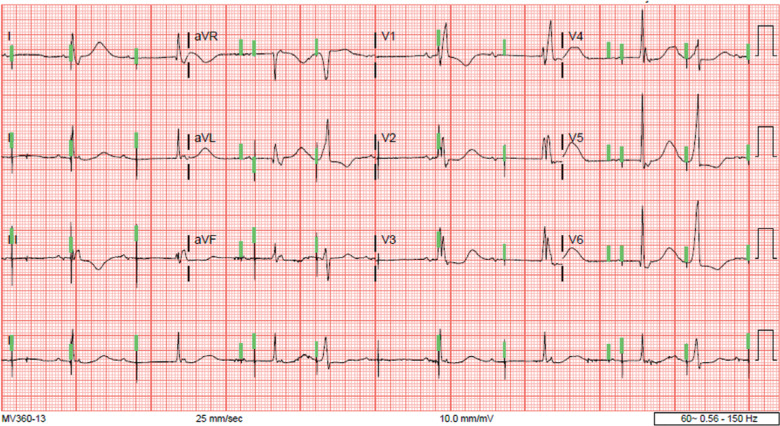
Marked bradycardia with intermittent failure to capture.

Following TAVR, inpatient telemetry monitoring for 24 h showed AV pacing without any loss of ventricular capture. A post-TAVR ECG showed loss of HB capture **(Figure 4)**. Interrogation of his PPM was performed 4 days post-TAVR, which showed an underlying complete heart block (CHB) with a loss of HB capture and an elevated RV capture threshold of >2.5 V @ 1 ms, and his RV outputs were increased. Then, 4 months later, the patient presented with an altered mental status with stroke-like symptoms and severe dizziness. A computed tomography scan of the brain was unremarkable for any ischemic or hemorrhagic changes. His ECG at that time, however, revealed marked bradycardia due to sinus rhythm with CHB with a slow escape and the same RBBB morphology as that seen at baseline with intermittent failure of ventricular capture from his PPM **(Figure 5)**. Interrogation of his PPM revealed diminished R-wave sensing of 1 mV and a significantly elevated RV pacing threshold of 5 V @ 1 ms, and his RV output was maximized to 7.5 V @ 1.5 ms. Given his dependence upon ventricular pacing, he was admitted to the hospital for RV lead revision.

**Figure 6:**
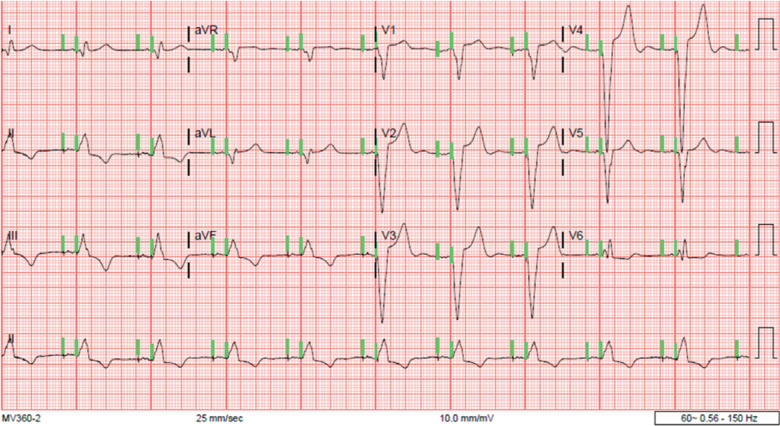
Atrioventricular paced rhythm.

During hospitalization, the patient developed a fever and worsening leukocytosis. Blood cultures revealed enterococcal bacteremia. Given the concern for endocarditis, a transesophageal echocardiogram was performed, which demonstrated an 8-mm mobile echo-density reflective of vegetations on the prosthetic aortic valve and the pacemaker lead with mild perivalvular leak, normal valve motion, and no echocardiographic evidence of a perivalvular abscess. The patient underwent explantation of the pacemaker and extraction of his pacing leads, and a temporary pacemaker was placed. After an adequate course of antibiotics with negative blood cultures for >72 h, he underwent successful re-implantation of a new right-sided Medtronic dual-chamber PPM. His RV lead was placed into the RV outflow tract septum. On follow-up, his ECG **(Figure 6)** revealed an appropriate AV paced rhythm and PPM interrogation confirmed normal device function with stable lead parameters.

The consent process was performed according to the recommendations from the investigational board review. A separate consent form was obtained.

## Discussion

TAVR is rapidly becoming the preferred treatment for patients with severe AS. Conduction abnormalities such as new-onset LBBB and AVB remain important complications that should be anticipated. Our patient developed CHB following TAVR, which manifested as a loss of HB capture. He also developed a progressive elevation of the local RV myocardial capture threshold well beyond the time frame that would have been attributed to acute local traumatic injury and inflammation. Despite the development of infective endocarditis of the prosthetic valve and pacing lead, we believe that the loss of HB capture was due to a direct traumatic injury to the HB distal to the HB pacing lead rather than the infection itself, given that the patient did not have any evidence of a perivalvular abscess. Due to his pre-existing RBBB, we hypothesize that traumatic injury occurred to the HB itself following TAVR, leading to CHB. Although the RBBB escape morphology was very similar to baseline, an additional AV nodal injury cannot be fully excluded. Interestingly, the origin of his escape complexes suggests a distal HB versus proximal left bundle rather than an AV nodal site of origin. Given that the HB passes through the membranous septum only a few millimeters beneath the non-coronary/right coronary cusp,^8,9^ the depth of the implantation becomes very important for patients with HB pacing, as deeper valve placement can significantly increase the risk of an HB injury and conduction disturbances. We also hypothesize that local inflammatory changes with subsequent scarring of the region around the lead tip most likely explain the increasing local myocardial capture. A previous report demonstrated edematous changes and increased leukocyte counts around the AV nodal region following valve implantation in patients who underwent TAVR.^10^

Physiologic pacing is increasingly becoming the preferred mode of ventricular pacing. The implant procedure has been made less challenging with the advent of new delivery sheaths and leads. The benefits of physiologic pacing include a lower risk of developing pacing-induced cardiomyopathy, heart failure, or mitral regurgitation.^6^ Some of the limitations of HB pacing include higher pacing thresholds both acutely and chronically, leading to early battery depletion and up to a 5% lead revision rate. Issues with lead sensing can lead to under- or over-sensing, which may inhibit pacing therapy.^11^

This case highlights the potential risk of pacemaker malfunction following a TAVR procedure in patients with an HB pacemaker. Care coordination with a structural interventional cardiologist to avoid implanting the TAVR too distal into the LVOT and attempting to land the distal struts of the TAVR proximal to the tip of the His pacing lead would be recommended. Modifications must be made to the clinical decision-making process prior to pacemaker implantation in patients with AVB and AS with the recommendation to avoid HB pacing therapy and opt for LBBAP as an alternative for providing physiologic pacing therapy.

## Conclusion

Conduction abnormalities may occur following TAVR. LBBAP could be an alternative to HB pacing to maintain physiologic pacing if TAVR is planned.
